# Methodological Quality Assessment of Meta-Analyses and Systematic Reviews of Probiotics in Inflammatory Bowel Disease and Pouchitis

**DOI:** 10.1371/journal.pone.0168785

**Published:** 2016-12-22

**Authors:** Jinpei Dong, Guigen Teng, Tiantong Wei, Wen Gao, Huahong Wang

**Affiliations:** Department of Gastroenterology, Peking University First Hospital, Peking University, Beijing, China; University Hospital Llandough, UNITED KINGDOM

## Abstract

**Background:**

Probiotics are widely used for the induction and maintenance of remission in inflammatory bowel disease (IBD) and pouchitis. There are a large number of meta-analyses (MAs)/ systematic reviews (SRs) on this subject, the methodological quality of which has not been evaluated.

**Objectives:**

This study aimed to evaluate the methodological quality of and summarize the evidence obtained from MAs/SRs of probiotic treatments for IBD and pouchitis patients.

**Methods:**

The PubMed, EMBASE, Cochrane Library and China National Knowledge Infrastructure (CNKI) databases were searched to identify Chinese and English language MAs/SRs of the use of probiotics for IBD and pouchitis. The Assessment of Multiple Systematic Reviews (AMSTAR) scale was used to assess the methodological quality of the studies.

**Results:**

A total of 36 MAs/SRs were evaluated. The AMSTAR scores of the included studies ranged from 1 to 10, and the average score was 5.81. According to the Canadian Agency for Drugs and Technologies in Health, 4 articles were classified as high quality, 24 articles were classified as moderate quality, and 8 articles were classified as low quality. Most of the MAs/SRs suggested that probiotics had potential benefits for patients with ulcerative colitis (UC), but failed to show effectiveness in the induction and maintenance of remission in Crohn’s disease (CD). The probiotic preparation VSL#3 may play a beneficial role in pouchitis.

**Conclusion:**

The overall methodological quality of the current MAs/SRs in the field of probiotics for IBD and pouchitis was found to be low to moderate. More MAs/SRs of high quality are required to support using probiotics to treat IBD and pouchitis.

## Introduction

Inflammatory bowel disease (IBD) encompasses a group of disorders of the gastrointestinal tract characterized by recurrent inflammation, with periods of relapse and remission, and epithelial injury [1]. IBD includes Crohn’s disease (CD) and ulcerative colitis (UC). The etiology of IBD is still unclear; however, there is some evidence to suggest that immune dysregulation, environmental factors, and genetic polymorphisms contribute to the multifactorial nature of the disease [2]. A dysbiosis of the intestinal microbiota also plays a role in the initiation or perpetuation of gut inflammation, which develops under an altered or impaired immune response [2].

In the hierarchy of evidence-based medicine, a meta-analysis (MA) or systematic review (SR) of high-quality randomized controlled trials (RCTs) is considered the best evidence for healthcare intervention [3]. High-quality MAs/SRs can aid policy makers in formulating guidelines and guide clinicians to make correct clinical decisions. In the past few decades, there have been many MAs/SRs that evaluated the role of probiotics in the course of treating IBD. Many reports have found that probiotics play a beneficial role in inducing and maintaining remission in mild-to-moderate UC but fail to show efficacy in CD. Unfortunately, the quality of these MAs/SRs had not been evaluated before probiotics were recommended. The objectives of this study were to use the Assessment of Multiple Systematic Reviews (AMSTAR) scale to evaluate the methodological quality of MAs/SRs of the role of probiotics in treating IBD and pouchitis patients, and summarize the published evidence to provide suggestions for making clinical decisions.

## Materials and Methods

### Data Sources and Study Selection

We searched for all MAs and SRs up to April 2016 using the PubMed, EMBASE, Cochrane Library and China National Knowledge Infrastructure (CNKI) databases. Combinations of the following keywords were used in the search: “inflammatory bowel disease”, “ulcerative colitis”, “Crohn’s disease”, “pouchitis”, “probiotics”, “*Lactobacillus*”, “*Bifidobacterium*”, “*Saccharomyces*”, “*Escherichia coli*” and “VSL#3”. The text words and MeSH terms were entered depending on the databases characteristics. The reference lists from retrieved articles were also screened for additional applicable studies. The language was restricted to Chinese and English.

### Inclusion and Exclusion Criteria

We included MAs/SRs that assessed probiotics as an intervention for the treatment of IBD and had full texts, regardless of publication status. Participants of any gender or ethnic origin with all types of IBD and pouchitis (once stated in the text) were eligible. We considered only adult IBD and pouchitis patients. Studies in an abstract form or meeting report were not considered. The protocols of the MAs/SRs were excluded.

### Study Selection

Two authors independently scanned the titles and abstracts of the MAs/SRs to identify eligible articles. If we could not judge whether an article was in keeping with the inclusion and exclusion criteria according to the citations of the search, we attempted to locate the full text. Any disagreement was resolved by discussion; a third reviewer was consulted if we could not reach a consensus.

### Assessment of Methodological Quality of Included MAs/SRs

Two authors independently assessed the methodological quality of the included MAs/SRs, using the AMSTAR scale [4], which comprises 11 methodological criteria.

These criteria include the following:

1. Was an a priori design provided?
2. Was there duplicate study selection and data extraction?
3. Was a comprehensive literature search performed?
4. Was the status of publication (i.e. grey literature) used as an inclusion criterion?
5. Was a list of studies (included and excluded) provided?
6. Were the characteristics of the included studies provided?
7. Was the scientific quality of the included studies assessed and documented?
8. Was the scientific quality of the included studies used appropriately in formulating conclusions?
9. Were the methods used to combine the findings of studies appropriate?
10. Was the likelihood of publication bias assessed?
11. Was the conflict of interest stated?

We judged each item as follows:

- Yes (when the criterion was explicitly met).
- No (when the criterion was explicitly not met).
- Can not answer (when the item was relevant but not described completely or not reported at all).
- Not applicable (when the item was not relevant).

Then, we calculated the quality scores: one point was given when the answer was “Yes”; otherwise, 0 points were given. According to the number of criteria met, the included articles were ranked into 3 levels: “high” (range 9–11), “moderate” (range 5–8), and “low” (range 0–4) [5]. Any disagreement was resolved by discussion, and a third reviewer was consulted if necessary.

## Results

### Study Identification

**Fig 1:**
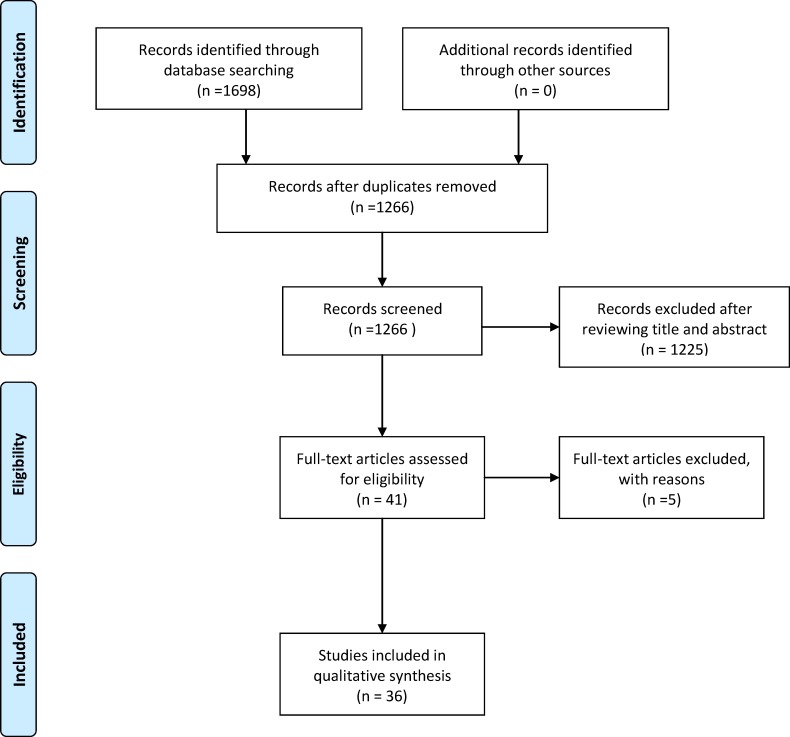
Flowchart of the review search and identification.

Overall, we identified 1698 potentially relevant articles by searching electronic databases and other resources. After reviewing the titles and abstracts and identifying duplications, 1657 articles were excluded. The remaining 41 articles were read in their entirety. Of these, 5 were excluded (four of the articles [6–9] were published in meeting abstracts, and we attempted to contact the authors to request the full text, but there was no reply; one article [10] was published in Polish). Finally, we included 36 studies in our analysis. The flowchart of the review selection process is presented in Fig 1.

### Characteristics of Included SRs

Among the included articles, 19 [11–29] (19/36, 52.8%) were published in English, and the other 17 [30–46] (17/36, 47.2%) were published in Chinese. There were 4 Cochrane reviews [21–24] and 2 academic dissertations [39, 42] among the 36 articles. The publication years of the 36 studies ranged from 2006 to 2016. The number of authors for the included articles ranged from 1 to 7, and the average number of authors was 3.83. The authors of the included articles searched 1 to 8 databases, and the studies involved contained a median of 4.83 databases.

The included articles covered 5 groups: UC (22/36, 61.1%) [12, 15, 20, 22, 24, 26, 29–39, 42–46], CD (5/36, 13.9%) [14, 16, 18, 21, 23], pouchitis (2/36, 5.6%) [13, 19], UC and CD (5/36, 13.9%) [11, 25, 27, 28, 40], and UC and CD and pouchitis (2/36, 5.6%) [17, 41]. The included 36 articles contained 443 studies, with an average of 12.3 studies per article. Regarding the probiotics reported in the included MAs/SRs, the following was found: one review [14] (1/36, 2.8%) evaluated the effect of *Lactobacillus*; one [29] (1/36, 2.8%) evaluated the effect of *Escherichia coli*; one [28] (1/36, 2.8%) evaluated the effect of *Lactobacillus and Bifidobacterium;* one [33] (1/36, 2.8%) evaluated the effect of *Bacillus subtilis* and *Enterococcus faecium*; one [45] (1/36, 2.8%) evaluated the effect of *Lactobacillus*, *Bifidobacterium* and *Streptococcus thermophiles;* two [20, 44] (2/36, 5.6%) evaluated the effect of VSL#3, while the remaining 29 (29/36, 80.6%) did not specify a category. Twenty-four (24/36, 66.7%) of the included articles reported side effects.

### Assessment of Methodological Quality of Included SRs

The methodological quality of the included articles was independently assessed by two authors using the AMSTAR tool. Any differences in their opinions were discussed and agreed upon via consensus. The assessments of methodological quality are described in Table 1. The average AMSTAR score of the included reviews was 5.81 (range 1–10), which indicates moderate quality. According to the AMSTAR scale and the rating system used by the Canadian Agency for Drugs and Technologies in Health (CADTH), only 4 of the MAs/SRs [21–24] were classified as high quality; 24 of the MAs/SRs [11, 13–18, 20, 26, 29–33, 35, 37–41, 43–46] were classified as moderate quality, and the other 8 MAs/SRs [12, 19, 25, 27, 28, 34, 36, 42] were classified as low quality. The most common AMSTAR items in which the reviews lost points were items 1, 4 and 5. The most common AMSTAR items to score points were items 6, 7, 9 and 10. For the other items, approximately half of the included MAs/SRs got the points. For item 7, the Jadad scale and Cochrane Collaboration’s tool were the most common tools used.

**Table 1:** Methodological quality of the included MAs/SRs.

**Item**	**Y, n (%)**	**N, n (%)**	**CA, n (%)**	**NA, n (%)**
**1. Was an 'a priori' design provided?**	4 (11.1%)	0 (0%)	32 (88.9%)	0 (0%)
**2. Was there duplicate study selection and data extraction?**	21 (58.3%)	0 (0%)	15 (41.7%)	0 (0%)
**3. Was a comprehensive literature search performed?**	19 (52.8%)	0 (0%)	17 (47.2%)	0 (0%)
**4. Was the status of publication used as an inclusion criterion?**	13 (36.1%)	7 (19.4%)	16 (44.4%)	0 (0%)
**5. Was a list of studies provided?**	6 (16.7%)	0 (0%)	30 (83.3%)	0 (0%)
**6. Were the characteristics of the included studies provided?**	26 (72.2%)	1 (2.8%)	9 (25.0%)	0 (0%)
**7. Was the scientific quality of the included studies assessed and documented?**	31 (86.1%)	5 (13.9%)	0 (0%)	0 (0%)
**8. Was the scientific quality of the included studies used appropriately in formulating conclusions?**	18 (50.0%)	18 (50.0%)	0 (0%)	0 (0%)
**9. Were the methods used to combine the findings of studies appropriate?**	30 (83.3%)	0(0%)	0(0%)	6 (16.7%)
**10. Was the likelihood of publication bias assessed?**	24 (66.7%)	12 (33.3%)	0 (0%)	0 (0%)
**11. Was the conflict of interest stated?**	17 (47.2%)	19 (52.8%)	0 (0%)	0 (0%)

Y: Yes, N: No, CA: Can not answer, NA: Not applicable

## Discussion

Evaluating the methodological quality of MAs/SRs is a means to evaluate how well the process of designing and conducting the research controlled for bias. The AMSTAR scale has been widely used since it was published in 2007, and this scale has become a recommended tool to evaluate the methodological quality of MAs/SRs [47].

The AMSTAR scores of the included reviews ranged from 1–10. Most of the articles were of poor or moderate quality; there were few high quality MAs/SRs. There were various defects regarding the information in the included articles. The main problems found in the MAs/SRs are described below.

First, there was no published protocol. To add scientific credibility and improve research standards, the protocol should be formulated prior to the MA/SR. A protocol may contribute to making the research process prospective, strict and transparent and may decrease the possibility that the authors will be influenced by the published articles regarding a certain process in conducting an MA/SR. In this overview, we found that only 4 MAs/SRs [21–24] had a published protocol, indicating that authors should pay more attention to this area in the future.

Second, the study selection and data extraction were not conducted by at least two authors independently. The AMSTAR scale claims that the search results and data extraction should be screened by at least two independent reviewers. This approach helps to prevent the inappropriate inclusion or exclusion of articles and thus reduces bias in the selection of studies [48]. Nearly half (15/36, 41.7%) of the included articles were not in accord this item or did not report clearly, which may increase the selection bias and decrease the quality of an MA/SR.

Third, a comprehensive literature search was not performed. Item 3 was primarily used to evaluate whether the search strategy was comprehensive. This item had two aspects; the first aspect was a comprehensive search plan, which contained keywords and retrieval types, and the other aspect was whether retrieval range was wide enough, containing at least two databases according to the AMSTAR scale. In addition, item 3 emphasized that supplemental searching is also of great significance. Nearly half (17/36, 47.2%) of the studies reported only the keywords used in searching electronic databases, and failed to consider the importance of a supplemental search.

Fourth, the status of publication was not used as an inclusion criterion. This item was mainly used to evaluate the selection bias. All authors and journals wish to publish articles with positive outcomes; articles with negative outcomes are either unpublished or published in informal journals. Selection bias may easily appear if the authors neglect gray literature.

Fifth, the MAs/SRs did not provide a list of both the included and excluded articles, which was a common problem as most studies provided only the included articles, and not both lists, possibly due to restricted page layouts. However, Cochrane SRs always provide lists of both the included and excluded articles, which allow the reader to easily judge the quality of the selected articles.

Sixth, the MAs/SRs did not consider whether the scientific quality of the included studies was used appropriately in formulating conclusions. This item required the results of the quality assessment and risk of bias to be considered in the analysis and conclusions of the review and to be explicitly stated in formulating recommendations [48]. Evidence-based medicine requires that research results are presented objectively and cautiously, rather than draw a tendentious conclusion hastily. This item requires that the quality of the included studies is sufficiently considered and reveals potential methodological flaws before reaching a conclusion.

Seventh, a conflict of interest statement was not included. This item is very important, especially in MAs/SRs involving pharmaceuticals. Research supported by pharmaceutical companies is more likely to obtain positive outcomes. If the author reports the conflict of interest in the MA/SR, readers may consider whether there is possibility of overvaluing the research results. In this overview, nearly half (17/36, 47.2%) of the included studies did not state conflict of interest.

We summarized the findings and evaluated the quality of the 36 included MAs/SRs as well as the number of RCTs and patients included in each MA/SR (Table 2). The more RCTs and patients included and the higher AMSTAR scores obtained, the more credible the conclusion is. Most of the included MAs/SRs focused on probiotics in UC, while only a few of the MAs/SRs evaluated the efficiency of probiotics in treating CD or pouchitis.

**Table 2:** Conclusions and the number of RCTs and patients included in each MA/SR and the AMSTAR score.

Conclusions	Number of included MAs/SRs	Average RCTs included	Average patients included	Average AMSTAR score
**Probiotics for the induction of remission in UC**				
The combination of probiotics with 5-aminosalicylates (5-ASA) is superior to 5-ASA alone in inducing remission in UC [20, 30, 33–35, 39, 43, 45]	8	9.9	689.4	6.38
No significant difference was observed between the curative effects of probiotics and 5-ASA in mild-to-moderate UC patients [22, 30, 35, 36, 39]	5	2.2	229.6	7.00
Probiotics are superior to a placebo in inducing remission in UC [11, 15, 17, 25, 26, 35, 36, 38, 42, 46]	10	4.5	304.2	5.50
The combination of probiotics with sulfasalazine is superior to sulfasalazine alone in inducing remission in UC [30, 43]	2	4.0	248.5	7.50
Probiotics are superior to the control group in inducing remission in UC [15, 27, 44]	3	6.3	429.0	4.33
**Probiotics for the maintenance of remission in UC**				
The administration of probiotics results in the same effect as 5-ASA in maintaining remission in UC [11, 12, 17, 24, 29, 31, 32, 35–42, 46]	16	4.1	507.9	5.94
The combination of probiotic agents with 5-ASA is superior to 5-ASA alone in maintaining remission in UC [34]	1	4.0	204.0	4.00
The combination of probiotic agents and 5-ASA is not superior to 5-ASA alone in preventing relapse [17, 35]	2	2.5	230.5	7.00
Probiotics are superior to a placebo in maintaining remission in UC [12, 26, 35–39]	7	2.6	70.3	5.57
**Probiotics for the induction of remission in CD**				
Probiotics are not superior to a placebo in inducing remission in CD [17]	1	3.0	74.0	8.00
**Probiotics for the maintenance of remission in CD**				
Probiotics are not superior to a placebo in maintaining remission in CD [14, 16–18, 23, 25, 27, 40, 41]	9	5.9	262.8	5.89
**Probiotics for the induction of remission in pouchitis**				
Probiotics play a beneficial role in achieving clinical improvement in pouchitis [13]	1	8.0	247.0	5.00
**Probiotics for the maintenance of remission in pouchitis**				
Probiotics are superior to a placebo in maintaining remission in pouchitis [19, 27, 41]	3	4.0	165.7	3.33
Probiotics are not superior to a placebo in maintaining remission in pouchitis [17]	1	4.0	148.0	8.00

A majority of the MAs/SRs concluded that UC patients benefit from probiotics; however, these studies failed to obtain a high AMSTAR score, which decreases the credibility of the conclusion. In examining the role of probiotics in the maintenance of remission in UC, two MAs [17, 35] concluded that the combination of probiotics and aminosalicylates is not superior to aminosalicylates alone in maintaining remission. However, another MA [34] drew a different conclusion, i.e., that the combination of probiotics and aminosalicylates is superior to aminosalicylates alone in maintaining a UC remission. Unfortunately, all of these studies included only a few RCTs and patients, and had a low to moderate AMSTAR score.

A few MAs/SRs evaluated the effectiveness of probiotics for CD, but most of these studies evaluated the role of probiotics in the maintenance of CD remission. Probiotics failed to show effectiveness in the induction and maintenance of remission in CD according to the current MAs/SRs, and the AMSTAR scores are variable, ranging from 1–10.

The included MAs/SRs reached different conclusions regarding the role of probiotics in maintaining remission in pouchitis. Three MAs/SRs [19, 27, 41] found that probiotics are superior to a placebo in maintaining a pouchitis remission, while another MA [17] drew the conclusion that patients with pouchitis did not gain an advantage in maintaining treatment with probiotics compared with a placebo. However, among the four RCTs included in the latter MA, three RCTs evaluated the role of VSL#3 in maintaining remission in pouchitis, and these three RCTs found that VSL#3 is beneficial for maintaining remission in patients with pouchitis. This finding suggested that the assessment of probiotics should be performed on a strain-specific basis and combining multiple strains for analysis may introduce a bias.

In addition, the disease activity index and the tools used should also be considered in evaluating the role of probiotics in IBD. Probiotics may play an effective role in inducing remission in mild-to-moderate UC, while in severe UC patients, probiotics may be less effective; a bias may thus be introduced if an author combines patients with different severities of UC in the analysis. Therefore, combining similar disease severities and strains of probiotics in subgroup analyses may be a good way to improve the credibility of conclusions. The evaluation of research depends on complete and accurate reporting. If the authors of clinical trials followed the Standards for Reporting Diagnostic accuracy studies (STARD) statement to report the eligibility criteria, diagnostic basis, baseline demographic and clinical characteristics, and distribution of disease severity among the participants, the trial would be much more useful and would provide higher quality comparisons.

Although many MAs/SRs concluded that the administration of probiotics results in additional benefit in UC patients, the Cochrane review, which is acknowledged as a high-quality SR, suggested that there is not enough evidence to recommend the use of probiotics for the treatment of UC, and this is an important point worth considering. Our work found that a majority of the 36 included articles were of low-to-moderate quality, which may influence the evidence level of applying probiotics to the treatment of IBD and pouchitis. In conclusion, we all benefit from more informative and high-quality MAs/SRs and require more high-quality MAs/SRs to support the use of probiotics in IBD.

## Supporting Information

S1 Text Search strategy of four databases. (DOCX)

S2 Text PRISMA 2009 checklist. (DOC)

S3 Text Relevant data underlying the findings described in the manuscript. (XLSX)

## References

[1] de Moreno de LeBlanc A, Del Carmen S. Current Review of Genetically Modified Lactic Acid Bacteria for the Prevention and Treatment of Colitis Using Murine Models. 2015;2015:146972. 10.1155/2015/146972 PMC4434185 26064086

[2] Basso PJ, Fonseca MT, Bonfa G, Alves VB, Sales-Campos H, Nardini V, et al Association among genetic predisposition, gut microbiota, and host immune response in the etiopathogenesis of inflammatory bowel disease. Brazilian journal of medical and biological research = Revista brasileira de pesquisas medicas e biologicas. 2014;47(9):727–37. 10.1590/1414-431X20143932 25075576 PMC4143199

[3] The Medical Research Library of Brooklyn. The Evidence Pyramid. 2004. Available from: http://library.downstate.edu/EBM2/2100.htm.

[4] Shea BJ, Grimshaw JM, Wells GA, Boers M, Andersson N, Hamel C, et al Development of AMSTAR: a measurement tool to assess the methodological quality of systematic reviews. BMC medical research methodology. 2007;7:10 10.1186/1471-2288-7-10 17302989 PMC1810543

[5] Canadian Agency for Drugs and Technologies in Health (CADTH). Interventions Directed to Consumers. 2011. Available from: www.cadth.caenresourcesrx-for-change.

[6] Abdollahi M, Nikfar S, Darvish Damavandi M. Efficacy of antibiotics and probiotics in management of pouchitis; a meta-analysis. Value in Health. 2010;13(7):A368–A9.

[7] Ashraf I, Sohail U, Arif M, Siddique S, Bechtold M, Choudhary A. Probiotic use for management of pouchitis: A systematic review and meta-analysis. American Journal of Gastroenterology. 2014;109:S677–S8.

[8] Doherty GA, Bennett G, Patil S, Cheifetz A, Moss AC. Meta-analysis of probiotics in the prevention of post-operative recurrence of Crohn's disease. Gastroenterology. 2009;136(5):A772–A3.

[9] Gordon M, Farrell M. Probiotics for maintenance of remission in ulcerative colitis: A cochrane systematic review. Inflammatory Bowel Diseases. 2016;22:S26–S7. 10.1097/MIB.0000000000000396 25844963

[10] Szajewska H, Horvath A, Dziechciarz P. Probiotics, prebiotics and synbiotics in the treatment of inflammatory bowel disease—A systematic review. Pediatria Wspolczesna. 2007;9(4):266–75.

[11] Fujiya M, Ueno N, Kohgo Y. Probiotic treatments for induction and maintenance of remission in inflammatory bowel diseases: a meta-analysis of randomized controlled trials. Clinical journal of gastroenterology. 2014;7(1):1–13. 10.1007/s12328-013-0440-8 26183502

[12] Rahimi R, Nikfar S, Rezaie A, Abdollahi M. A meta-analysis of the benefit of probiotics in maintaining remission of human ulcerative colitis: Evidence for prevention of disease relapse and maintenance of remission. Archives of Medical Science. 2008;4(2):185–90.

[13] Nikfar S, Darvish-Damavandi M, Abdollahi M. A review and meta-analysis of the efficacy of antibiotics and probiotics in management of pouchitis. International Journal of Pharmacology. 2010;6(6):826–35.

[14] Shen J, Ran HZ, Yin MH, Zhou TX, Xiao DS. Meta-analysis: The effect and adverse events of Lactobacilli versus placebo in maintenance therapy for Crohn disease. Internal Medicine Journal. 2009;39(2):103–9. 10.1111/j.1445-5994.2008.01791.x 19220543

[15] Zigra PI, Maipa VE, Alamanos YP. Probiotics and remission of ulcerative colitis: A systematic review. Netherlands Journal of Medicine. 2007;65(11):411–8. 18079563

[16] Rahimi R, Nikfar S, Rahimi F, Elahi B, Derakhshani S, Vafaie M, et al A meta-analysis on the efficacy of probiotics for maintenance of remission and prevention of clinical and endoscopic relapse in Crohn's disease. Digestive diseases and sciences. 2008;53(9):2524–31. 10.1007/s10620-007-0171-0 18270836

[17] Shen J, Zuo ZX, Mao AP. Effect of probiotics on inducing remission and maintaining therapy in ulcerative colitis, Crohn's disease, and pouchitis: meta-analysis of randomized controlled trials. Inflamm Bowel Dis. 2014;20(1):21–35. 10.1097/01.MIB.0000437495.30052.be 24280877

[18] Doherty GA, Bennett GC, Cheifetz AS, Moss AC. Meta-analysis: targeting the intestinal microbiota in prophylaxis for post-operative Crohn's disease. Alimentary pharmacology & therapeutics. 2010;31(8):802–9. 20055785 10.1111/j.1365-2036.2010.04231.x

[19] Elahi B, Nikfar S, Derakhshani S, Vafaie M, Abdollahi M. On the benefit of probiotics in the management of pouchitis in patients underwent ileal pouch anal anastomosis: a meta-analysis of controlled clinical trials. Digestive diseases and sciences. 2008;53(5):1278–84. 10.1007/s10620-007-0006-z 17940902

[20] Mardini HE, Grigorian AY. Probiotic mix VSL#3 is effective adjunctive therapy for mild to moderately active ulcerative colitis: a meta-analysis. Inflamm Bowel Dis. 2014;20(9):1562–7. 10.1097/MIB.0000000000000084 24918321

[21] Butterworth AD, Thomas AG, Akobeng AK. Probiotics for induction of remission in Crohn's disease. The Cochrane database of systematic reviews. 2008;(3):Cd006634 10.1002/14651858.CD006634.pub2 18646162 PMC6544811

[22] Mallon P, McKay D, Kirk S, Gardiner K. Probiotics for induction of remission in ulcerative colitis. The Cochrane database of systematic reviews. 2007;(4):Cd005573 10.1002/14651858.CD005573.pub2 17943867

[23] RolfeVE, FortunPJ, HawkeyCJ, Bath-HextallF. Probiotics for maintenance of remission in Crohn's disease. The Cochrane database of systematic reviews. 2006;(4):Cd004826 10.1002/14651858.CD004826.pub2 17054217

[24] Naidoo K, Gordon M, Fagbemi AO, Thomas AG, Akobeng AK. Probiotics for maintenance of remission in ulcerative colitis. The Cochrane database of systematic reviews. 2011;(12):Cd007443 10.1002/14651858.CD007443.pub2 22161412

[25] Jonkers D, Penders J, Masclee A, Pierik M. Probiotics in the management of inflammatory bowel disease: a systematic review of intervention studies in adult patients. Drugs. 2012;72(6):803–23. 10.2165/11632710-000000000-00000 22512365

[26] Sang LX, Chang B, Zhang WL, Wu XM, Li XH, Jiang M. Remission induction and maintenance effect of probiotics on ulcerative colitis: a meta-analysis. World journal of gastroenterology. 2010;16(15):1908–15. 10.3748/wjg.v16.i15.1908 20397271 PMC2856834

[27] Ghouri YA, Richards DM, Rahimi EF, Krill JT, Jelinek KA, DuPont AW. Systematic review of randomized controlled trials of probiotics, prebiotics, and synbiotics in inflammatory bowel disease. Clinical and experimental gastroenterology. 2014;7:473–87. 10.2147/CEG.S27530 25525379 PMC4266241

[28] Saez-Lara MJ, Gomez-Llorente C, Plaza-Diaz J, Gil A. The role of probiotic lactic acid bacteria and bifidobacteria in the prevention and treatment of inflammatory bowel disease and other related diseases: a systematic review of randomized human clinical trials. BioMed research international. 2015;2015:505878 10.1155/2015/505878 25793197 PMC4352483

[29] Losurdo G, Iannone A, Contaldo A, Ierardi E, Di Leo A, Principi M. Escherichia coli Nissle 1917 in Ulcerative Colitis Treatment: Systematic Review and Meta-analysis. Journal of gastrointestinal and liver diseases: JGLD. 2015;24(4):499–505. 10.15403/jgld.2014.1121.244.ecn 26697577

[30] Dai ZJ, Tan QH, Li XH. Effect of probiotics on inducing remission of mild to moderate ulcerative colitis in China: a Meta-analysis. Chin J Clinicians (Electronic Edition). 2014;(07):1303–8.

[31] Lu J, Wu JX. Evaluation of Probiotics on the Maintenance of Remission in Ulcerative Colitis. J Clin Intern Med. 2009;26(1):32–5.

[32] Niu XP, Han Z, Liu SF. The effect of probiotics in maintaining remission of ulcerative colitis: a meta analysis. Chin J Microbiol. 2010;22(3):261–3.

[33] Hu ZB, Chen LJ, Teng F, Liu LL, Lu HB. Systematic Evaluation on Medilac-S Combined Conventional Treatment for Ulcerative Colitis. Chin Pharm. 2013;22(7):3–7.

[34] Chen RH, Li YF, Yang X, Xia B. Therapeutic Effect of Probiotic Agents on Ulcerative Colitis: A Systematic Review. Chin J Gastroenterol. 2012;17(4):221–5.

[35] Wu QR, Zhou J, He JD, Wang YP, Yang XR, Zhang L. Probiotic Agents for Ulcerative Colitis: A Systematic Review. Chin J Evid-based Med. 2008;8(5):315–21.

[36] Ye K, Wang HL, Liao WF, Zeng FY. Probiotics as an ulcerative colitis in remission induction and drug maintenance treatment: a meta-analysis. J China-Japan Friendship Hosp. 2014;28(5):288–92.

[37] Ma JC, Zhang XL. Efficacy of probiotic agents in maintaining remission of ulcerative colitis: a meta analysis. Shi Jie Hua Ren Xiao Hua Za Zhi. 2008;16(36):4123–7.

[38] Tang SH, Feng SF, Yao YF. Effect of probiotic preparations on inducing and mainfaining remission of ulcerative colitis:a meta-analysis. Med J Chin PLA. 2010;35(5):521–5.

[39] Liao ZH. A Meta-analysis for the Treatment Effect of Probioties on Induction of Remission and Maintenance in Patients with Ulcerative Colitis. Thesis, Jinan university. 2010. Available from: http://www.cnki.net/KCMS/detail/detail.aspx?QueryID=2&CurRec=2&recid=&filename=2010124555.nh&dbname=CMFD2010&dbcode=CMFD&pr=&urlid=&yx=&uid=WEEvREcwSlJHSldTTGJhYlNiM3pYYUFuOXFFNndBZmNpaWtrNVJUODR2RGk=$9A4hF_YAuvQ5obgVAqNKPCYcEjKensW4ggI8Fm4gTkoUKaID8j8gFw!!&v=MTQwMThNMUZyQ1VSTHlmWU9ac0ZDbm1VN3JJVjEyNkhySzZHdFRKcXBFYlBJUjhlWDFMdXhZUzdEaDFUM3FUclc=.

[40] Wang XT, Dai JF, Lv B. Probiotics for Induction and Maintenance of Remission in Inflammatory Bowel Disease: A Meta-analysis. Chin J Gastroenterol. 2015;20(1):29–35.

[41] Shen J, Ran ZH, Tong JL, Xiao SD. Effect of probiotics on remission, relapse and pouchitis in inflammatory bowel disease: meta-analysis. Chin J Gastroenterol Hepatol. 2008;17(2):114–8.

[42] Ma XF. Effects of probiotics on remission and relapse in adult ulcerative colitis A meta-analysis. Thesis, Nanjing University of Chinese Medicine. 2013. Available from: http://www.cnki.net/KCMS/detail/detail.aspx?QueryID=0&CurRec=1&recid=&filename=1014416309.nh&dbname=CMFD201501&dbcode=CMFD&pr=&urlid=&yx=&uid=WEEvREcwSlJHSldTTGJhYlNiM3pYYUFuOXFFNndBZmNpaWtrNVJUODR2RGk=$9A4hF_YAuvQ5obgVAqNKPCYcEjKensW4ggI8Fm4gTkoUKaID8j8gFw!!&v=MTEwMjBGckNVUkx5ZllPWnNGQ25tVmI3SlZGMjZHcmU1R05MTXBwRWJQSVI4ZVgxTHV4WVM3RGgxVDNxVHJXTTE=.

[43] Li XM, Li X, Pu FF, Shi L, Hu W. Meta-analysis of the effectiveness of using probiotics in conjunction with aminosalicylic acid on remission induction of ulcerative colitis. Morden Prevent Med. 2014;(24):4439–42.

[44] Ding J, Xiong GS, Yang CH, Wu JH. Probiotic Preparation VSL#3 for Induction of Remission in Ulcerative Colitis: A Systematic Review. Chin J Gastroenterol. 2012;(09):521–6.

[45] Jin DC, Lu DR, Zhang BJ. Treatment of ulcerative colitis with golden bifid combined with aminosalicylates: a Meta-analysis. Chin J Clinicians(Electronic Edition). 2016;(03):401–5.

[46] Zhang QN, Huo LJ, Luo RL, Zhang J. Efficacy and safety of probiotics on mild and moderate activity and remission ulcerative colitis: a meta analysis. Contemporary Med. 2016;(05):4–7.

[47] Cornell JE, Laine C. The science and art of deduction: complex systematic overviews. Annals of internal medicine. 2008;148(10):786–8. 18490692 10.7326/0003-4819-148-10-200805200-00012

[48] Sharif MO, Janjua-Sharif FN, Ali H, Ahmed F. Systematic reviews explained: AMSTAR-how to tell the good from the bad and the ugly. Oral health and dental management. 2013;12(1):9–16. 23474576

