# Supplementary material for: Methodological Quality Assessment of Meta-Analyses and Systematic Reviews of Probiotics in Inflammatory Bowel Disease and Pouchitis
Source: PLoS One. 2016 Dec 22;11(12):e0168785. doi: 10.1371/journal.pone.0168785 (PMC5179087; doi:10.1371/journal.pone.0168785)
Supplement: S1 Text — (DOCX) [file pone.0168785.s001.docx]

**Search strategy of Pubmed**

#1 Search "inflammatory bowel disease" OR "ulcerative colitis" OR "Crohn disease" OR pouchitis

#2 Search probiotics OR Lactobacillus OR Bifidobacterium OR Saccharomyces OR Escherichia coli OR VSL

#3 Search ((("Inflammatory Bowel Diseases"[Mesh]) OR "Colitis, Ulcerative"[Mesh]) OR "Crohn Disease"[Mesh]) OR "Pouchitis"[Mesh]

#4 Search (((("Probiotics"[Mesh]) OR "Lactobacillus"[Mesh]) OR "Bifidobacterium"[Mesh]) OR "Saccharomyces"[Mesh]) OR "Escherichia coli"[Mesh]

#5 Search (#1) OR #3

#6 Search (#2) OR #4

#7 Search (#)5 AND #6

#8 Search (#5) AND #6 Filters: Meta-Analysis; Systematic Reviews

**Search strategy of Embase**

#1 'inflammatory bowel disease'/exp OR 'ulcerative colitis'/exp OR 'crohn disease'/exp OR 'ileitis'/exp

#2 'inflammatory bowel disease' OR 'ulcerative colitis' OR 'crohn disease' OR ileitis

#3 'probiotic agent'/exp OR 'Lactobacillus'/exp OR 'Bifidobacterium'/exp OR 'Saccharomyces'/exp OR 'Saccharomyces'/exp OR 'Escherichia coli'/exp OR 'Escherichia coli'/exp OR 'VSL3'/exp

#4 probiotics OR lactobacillus OR bifidobacterium OR saccharomyces OR escherichia OR vsl

#5 #1 OR #2

#6 #3 OR #4

#7 #5 AND #6

#8 #7 AND ('meta analysis'/de OR 'systematic review'/de)

**Search strategy of Cochrane Library**

#1 MeSH descriptor: [Probiotics] explode all trees

#2 MeSH descriptor: [Lactobacillus] explode all trees

#3 MeSH descriptor: [Bifidobacterium] explode all trees

#4 MeSH descriptor: [Saccharomyces] explode all trees

#5 MeSH descriptor: [Escherichia] explode all trees

#6 #1 or #2 or #3 or #4 or #5

#7 MeSH descriptor: [Inflammatory Bowel Diseases] explode all trees

#8 MeSH descriptor: [Colitis, Ulcerative] explode all trees

#9 MeSH descriptor: [Crohn Disease] explode all trees

#10 MeSH descriptor: [Pouchitis] explode all trees

#11 #7 or #8 or #9 or #10

#12 "inflammatory bowel disease" or "ulcerative colitis" or "Crohn disease" or pouchitis

#13 probiotics or Lactobacillus or Bifidobacterium or Saccharomyces or Escherichia coli or VSL

#14 #6 or #13

#15 #11 or #12

#16 #14 and #15 in Cochrane Reviews (Reviews only) and Other Reviews

**Search strategy of CNKI**

(FT=’益生菌’ OR FT=’乳酸杆菌’ OR FT=’酵母菌’ OR FT=‘双歧杆菌’ OR FT=’大肠杆菌’ OR FT=’VSL#3’) AND (FT=’炎症性肠病’ OR FT=’溃疡性结肠炎’ OR FT=’克罗恩病’ OR FT=’贮袋炎’) AND (FT=’Meta分析’ OR FT=’系统评价’ OR FT=’荟萃分析’ FT=’系统综述’)
